# Detection of *Chlamydophila pneumoniae* and human herpesvirus 8 in primary cutaneous anaplastic large-cell lymphoma: a case report

**DOI:** 10.1186/1750-9378-8-41

**Published:** 2013-10-07

**Authors:** Alessandro Borghi, Elisabetta Caselli, Dario Di Luca, Adolfo Sebastiani, Paolo Perri, Silva Seraceni, Carlo Contini, Annarosa Virgili

**Affiliations:** 1 Department of Medical Sciences, Section of Dermatology, University of Ferrara, Via Savonarola 9, 44121 Ferrara, Italy; 2 Department of Medical Sciences, Section of Microbiology, University of Ferrara, Ferrara, Italy; 3 Department of Ophthalmology, University of Ferrara, Ferrara, Italy; 4 Department of Medical Sciences, Section of Infectious Diseases, University of Ferrara, Ferrara, Italy

**Keywords:** Primary cutaneous anaplastic large-cell lymphoma, *Chlamydophila pneumoniae*, Human herpesvirus 8, Infection-related carcinogenesis, Eyelid

## Abstract

**Background:**

The etiology of primary cutaneous anaplastic large-cell CD30+ lymphoma is largely unknown, and although an infectious involvement has been suspected, the implication of infectious agents in its pathogenesis is still unclear.

**Findings:**

We report the case of a HIV-negative patient referred to our hospital with a rapidly enlarging skin tumor on her upper eyelid. Surgical excision was performed and histological analysis evidenced a primary cutaneous anaplastic large-cell lymphoma. Due to the ocular localization and to the prominent angiogenic component of the lesion, molecular analyses for the detection of *Chlamydophila pneumonia*e and HHV8 were performed, revealing the presence of an infection by both pathogens in surgical biopsy and in peripheral blood mononuclear cells.

**Conclusions:**

These findings suggest for the first time a possible association of *C. pneumoniae* and/or HHV8 infection, or both together, with primary cutaneous anaplastic large-cell lymphoma in non-immunocompromised and HIV-negative subjects. This potential pathogenic association, if confirmed, could provide potential indications for future therapy.

## Introduction

The risk factors for the development of cutaneous lymphomas are largely unknown. An infectious etiology has been suspected and investigated [1], but the implication of infectious agents in the pathogenesis of cutaneous lymphoproliferative disorders is still controversial.

*Chlamydophila pneumoniae* is an obligate intracellular pathogen with a worldwide diffusion and high seroprevalence in the adult population (50-70%) [2]. Although *C. pneumoniae* is known to infect human keratinocytes *in vitro* and to be associated with some cutaneous T cell lymphomas (CTCLs) [3,4], its implication in the pathogenesis of skin lymphomas remains unclear.

Human herpesvirus 8 (HHV8) prevalence varies geographically [5], being significantly higher in HIV-infected patients [6,7]. HHV8 is causally related to KS and is associated to other malignancies, including Multicentric Castleman’s Disease (MCD), Primary Effusion Lymphomas (PEL) and other lymphoproliferative disorders in immunosuppressed patients [8]. *In vivo*, HHV8 has been found in KS endothelial cells, in T and B lymphocytes, monocytes and keratinocytes, showing a broad cell tropism [9].

Here we report the case of an immunocompetent patient with a primary cutaneous anaplastic large-cell lymphoma (PCALCL) characterized by the presence of active *C. pneumoniae* and HHV8 infections.

### Case report

**Figure 1:**
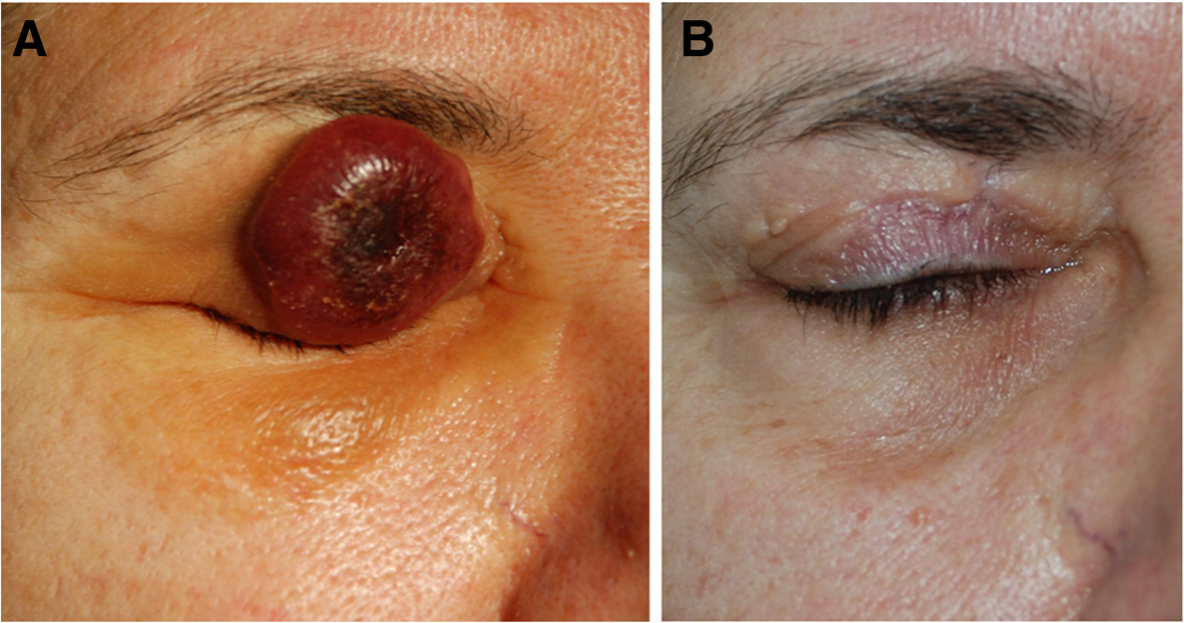
**Clinical features. (A)** The skin tumor on patient’s upper right eyelid; **(B)** No relapse occurred 12 months after surgical excision.

A 64-year-old woman presented at our Dermatology outpatient Unit with a 6-week-history of a single, painless, 26 mm in diameter rapidly enlarging skin tumor located on her upper right eyelid (Figure 1). The lesion was elevated, firm, of reddish-brown color, with well-defined borders and central crater-like ulcero-necrotic depression covered with crust. No ocular involvement was assessed. Physical examination was unremarkable and there was no palpable lymphadenopathy. The patient’s medical history included Hashimoto’s thyroiditis long-term treated with levothyroxine and chronic urticaria cyclically treated with oral antihistamine. At the onset, the lesion had been treated with aspecific systemic and topical antibiotics in the suspect of a pyodermitis, but the clinical picture worsened. The patient complained of about 2-week slight intermittent fever, sweats, and itching but without weight loss.

## Methods and results

**Figure 2:**
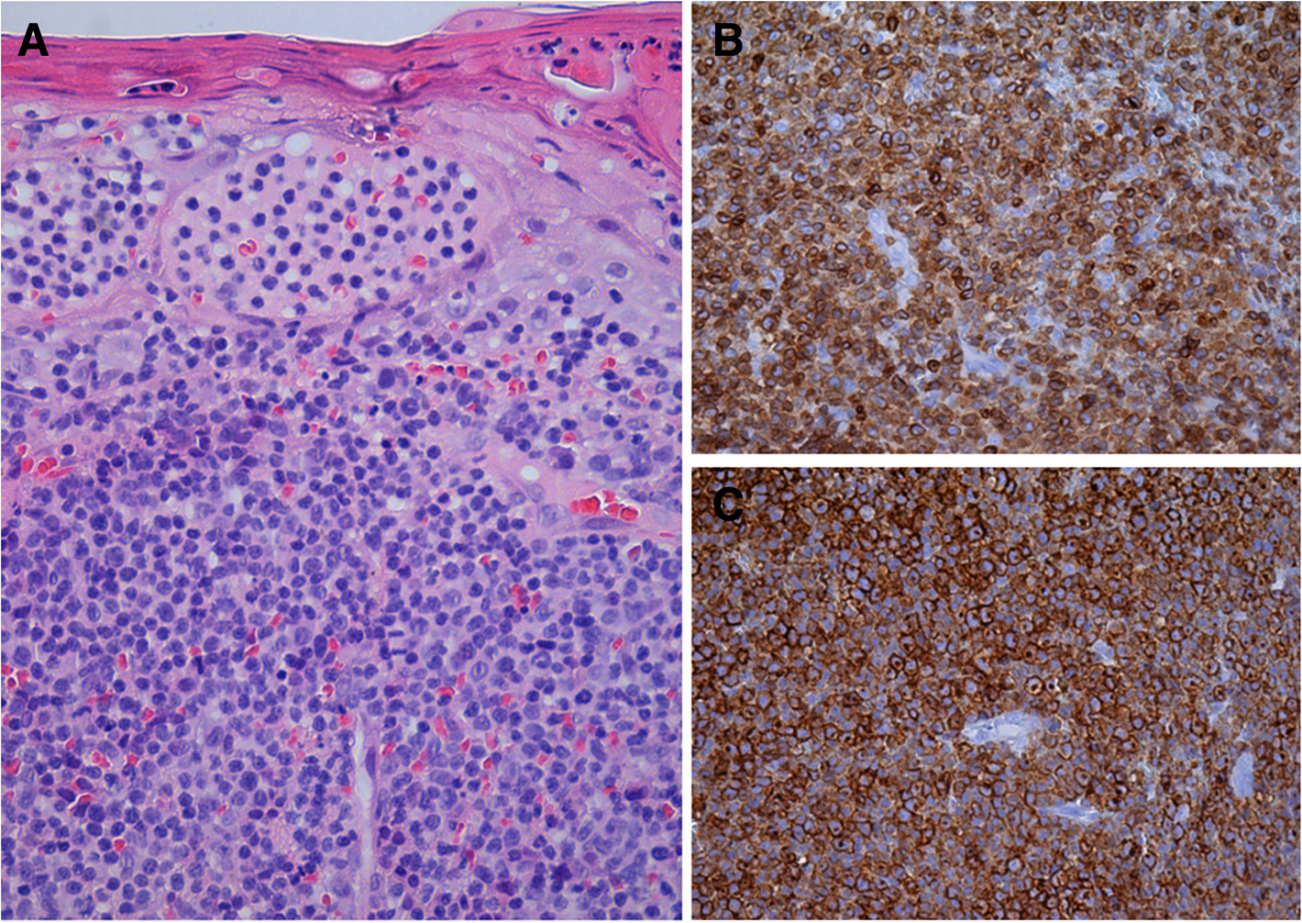
**Histological and immunohistochemical findings. (A)** Diffuse lymphocytic infiltration of the dermis predominantly composed of large pleomorphic cells with irregular nuclei and prominent nucleoli (hematoxylin-eosin staining, magnification 20×). By immunoperoxidase staining, the neoplastic cells showed marked and diffuse expression of CD3 **(B)** and CD30 **(C)**.

A surgical excision of the lesion was performed at the Ophthalmological Unit and histological examination revealed diffuse lymphocytic infiltration of the dermis with anaplastic lymphoid large cells (Figure 2). Tumor cells presented irregular nucleus, frequent mitotic figures, and were positive for CD3, CD2 and CD30, and negative for CD5, CD56, ALK and CD20, as determined by immunohistochemistry. The Ki67/MIB-1 proliferation index was about 90%. BIOMED-2 molecular analysis evidenced monoclonal rearrangement of the TCR-γ gene. Systemic evaluation including a positron emission tomography/computed tomography scan of the body, bone marrow biopsy, complete blood count with differential, and blood chemistry including lactate dehydrogenase, showed no evidence of extracutaneous involvement. Orbital magnetic resonance imaging also gave normal results. The patient’s serum was negative for anti-HIV antibodies. The diagnosis was primary cutaneous CD30+ anaplastic large cell lymphoma.

Considering the ocular localization and the histological features of the lesion, and the high potential of Chlamydia and HHV8 in lymphoma induction, the local and systemic presence of both pathogens was investigated, after receiving informed consent from the patient. The research has been carried out in compliance with the Helsinki Declaration. Approval by the institutional review board of our Hospital was not required for the present study, as materials were not collected specifically for this study, and were completely de-identified.

**Figure 3:**
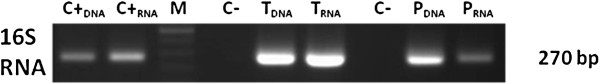
***C. pneumoniae *detection in tumor biopsy (T) and PBMCs (P).** Total DNA and RNA were extracted by tissue specimen and PBMC and analyzed by PCR and RT-PCR for 16s RNA *C*. *pneumoniae* gene. Amplification controls include negative (C-) and positive controls (corresponding respectively to DNA and RNA extracted from *C. pneumoniae* TW183 strain); Marker 100 bp (M).

*C. pneumoniae* was searched in cutaneous biopsy and in peripheral blood mononuclear cells (PBMCs) isolated by density gradient from peripheral blood (Fycoll-paque plus, GE Healthcare Europe GmbH, Milan, Italy). DNA and RNA were extracted as previously described [10,11], and assayed by PCR. A fraction of PBMCs was instead centrifuged, suspended in RPMI 1640 medium (Gibco, Invitrogen, Carlsbad, CA) and co-cultured up to 144 hours with Hep-2 cell line (ATCC CCL-23), to increase the number of bacterial inclusions [11]. Cultured samples were then processed as done for fresh uncultured cells. Analyses included nested-PCR and PCR after retrotranscription (RT-PCR), using primer sets targeting 16S rRNA, outer membrane protein (ompA/MOMP), and HsP-60 genomic regions of *C. psittaci*, *C. pneumoniae*, and *C. trachomatis*[11]. Amplification fragments, corresponding to *C. pneumoniae* 16S rRNA gene, were found both in tumor tissue and in co-cultured PBMCs (Figure 3). Amplicons were sequenced by ABI PRISM 377 DNA Sequencer (Applied Biosystems, The Netherlands) and compared with other *Chlamydiaceae* genes [12], showing a strict homology with *C. pneumoniae* TW183 strain (AE009440.1, 99%; E value: < 0.01).

**Figure 4:**
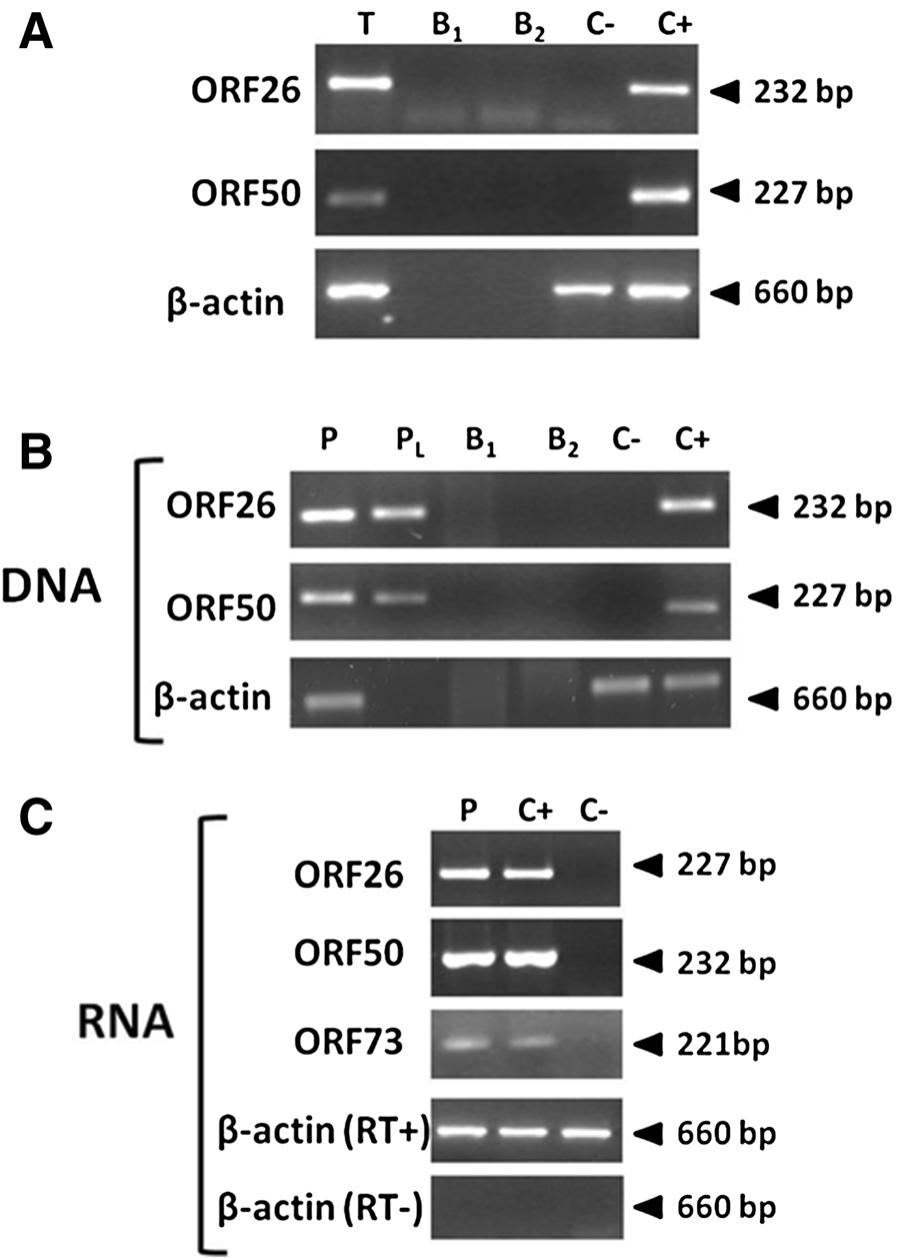
**HHV8 presence in tumor biopsy and peripheral blood. (A)** Total DNA extracted from biopsy tissue (T) was analyzed by PCR for HHV8 ORF50 and ORF26 genes. Amplification of the house-keeping β-actin gene was included as a control, and DNA from a T cell line negative for HHV8 (JJhan cells) and from a PEL-derived B cell line latently infected by HHV8 (BCBL-1 cells) were respectively used as negative (C-) and positive (C+) controls. Amplification controls also included: blanks of extraction (B_1_) and amplification (B_2_). **(B)** Total DNA extracted from PBMCs (P) and plasma (P_L_) fractions were analyzed as described for tumor biopsy. **(C)** Total RNA extracted from PBMCs was analyzed by RT-PCR for HHV8 lytic (ORF50, ORF26) and latent (ORF73) genes. Negative (C-) and positive (C+) controls, and β-actin control with (RT+) or without (RT-) previous retrotranscription are also shown.

In parallel, the same specimens were also used for HHV8 search, by PCR or real time quantitative PCR (qPCR) specifically designed in three different regions of HHV8 ORF50 and ORF26 genes [10]. The analysis of three different gene segments was undertaken to avoid the risk of false positive/negative results connected with a single gene detection. Results showed that both tumor biopsy and PBMCs harbored HHV8 sequences (Figure 4A-B). Quantification of viral load, as determined by qPCR, showed 1.24 × 10^3^ virus genome copies per 100 ng total DNA in tumor biopsy and 5 × 10^3^ copies/100 ng DNA in PBMCs. HHV8 DNA was detected also in the plasma fraction of peripheral blood (Figure 4B), suggesting the presence of virions released from productively infected cells. Consistently with this, transcriptional analysis of HHV8 in PBMCs showed the presence of lytic ORF50/ORF26 transcripts, in addition to latent ORF73 mRNA, demonstrating that HHV8 was actively replicating, as ORF50/ORF26 genes are only expressed during the productive phase of infection (Figure 4C). Since EBV infection has been associated to the occurrence of ocular lymphomas, the specimens were also analyzed for the presence of EBV, by specific nested PCR, obtaining negative results.

Due to the detection of active chlamydial infection, the patient was given doxycycline 100 mg, twice a day, for 4 weeks. This treatment was safe and well tolerated, and *C. pneumoniae* was no longer detectable in PBMCs after the conclusion of antibiotic treatment.

Follow up analyses were performed after 12 months, during which the patient has remained free of skin and systemic disease. *C. pneumoniae* was no longer detectable in the patient’s PBMCs, and HHV8 was no longer sustaining active infection, suggesting that the virus entered the latent phase.

## Discussion

Primary cutaneous CD30+ lymphoproliferative disorders are the second most common form of CTCLs, including PCALCL [13]. PCALCL usually occur as rapidly growing and ulcerating large tumors, histologically characterized by large tumor cells with an anaplastic, pleomorphic, and immunoblastic cytomorphology, largely expressing (>75%) CD30. In the etiology of CTCLs, a role for superantigenic activation of T cells leading to accumulation of skin-homing T cells has been suggested [14], and defects in apoptosis signalling in skin-homing T cells have been hypothesized to play a role [15]. However conclusive data are still lacking.

Accumulating evidence suggests that Chlamydia Spp. may play a role in oncogenesis for their tendency to cause persistent infections and chronic antigenic stimulation. *C. psittaci* has been associated to ocular adnexal lymphomas (OALs) of MALT-type [3]. *C. pneumoniae* has been shown to be present in some CTCL and to induce the expansion of *C. pneumoniae*-specific T cells throughout the release of Sézary T cell-activating factor (SAF), thus potentiating the development of CTCL [4]. *C. pneumoniae* eradication with doxycycline has been reported to result in lymphoma regression in about a half of patients [16], as also evidenced for *C. trachomatis*, supposed to have a role in ocular lymphoma [17]. Moreover, chlamydial infection has been detected in some forms of cutaneous lymphomas and in diffuse large B-cell lymphomas arising in the Waldeyer’s ring [3].

On the other hand, several viruses have been investigated as possible trigger agents of CTCLs, including herpesviruses. EBV infection was associated to extranodal NK/T-cell lymphoma and some primary cutaneous lymphomas [18]. Cytomegalovirus infection was associated with CTCLs [19], but the association is still controversial.

HHV8 was found in various tumors and lymphoproliferative diseases [8], and the incidence of HHV8-related tumors, even in populations with high HHV8 seroprevalence suggests the need for cofactors [20]. A number of HHV8 genes are known to have effects in cell proliferation and transformation [8], and accumulating data point to the importance of lytic replication for the development of HHV8-associated neoplastic pathologies, as indicated by recent clinical studies [21]. Our data present evidence of a concurrent active infection by *C. pneumoniae* and HHV8 in a PCALCL patient, suggesting for the first time a potential association of chlamydial and HHV8 infection with the development of the PCALCL. Although the possibility exists that it might be simply accidental, the presence of both microorganisms is intriguing, and suggests that they might reciprocally potentiate their pathogenic potential. This hypothesis has been suggested for other pathologies without obtaining clear conclusions [22,23], and may deserve further investigation. In parallel, individual susceptibility might be important for the development of the disease. We recently reported that specific Killer Inhibitory Receptors (KIR) present on NK cells are related to a decreased ability to control herpesviruses infection [24], and it may be hypothesized that individuals with an immune response not capable of controlling infectious replication might be more susceptible to infection-related tumor development.

Any consideration about the possible role of antibiotic therapy in treating the neoplasm cannot be drawn, as the antibiotic treatment was started when the tumor had been surgically excised.

Studies of more patients would be needed to clarify the role of pathogens in PCALCL development.

## Abbreviations

PCALCL: Primary cutaneous anaplastic large-cell lymphoma; CTCLs: Cutaneous T cell lymphomas; HHV8: Human herpesvirus 8; EBV: Epstein-Barr virus; MCD: Multicentric Castleman’s Disease; PEL: Primary Effusion Lymphomas; PBMCs: Peripheral blood mononuclear cells.

## Competing interest

The authors declare that they have no competing interests.

## Authors’ contributions

AB contributed to: study concept and design, acquisition of data (clinical features), analysis and interpretation of data, drafting of the manuscript. EC contributed to: study concept and design, acquisition of data (HHV8 analyses), analysis and interpretation of data, drafting of the manuscript. DDL contributed to: study concept and design, analysis and interpretation of data. AS contributed to: acquisition of data (clinical parameters, surgery), analysis and interpretation of data. PP contributed to: surgery, analysis and interpretation of data. SS contributed to: acquisition of data (Chlamydia analyses), interpretation of data. CC contributed to: study concept and design, acquisition of data (Chlamydia analyses), analysis and interpretation of data, drafting of the manuscript. AV contributed to: study concept and design, acquisition of data (clinical and histological features), analysis and interpretation of data. All authors read and approved the final manuscript.
